# Unmet need for family planning among reproductive-age women living with HIV in Ethiopia: A systematic review and meta-analysis

**DOI:** 10.1371/journal.pone.0255566

**Published:** 2021-08-02

**Authors:** Bereket Kefale, Bezawit Adane, Yitayish Damtie, Mastewal Arefaynie, Melaku Yalew, Assefa Andargie, Elsabeth Addisu

**Affiliations:** 1 Department of Reproductive and Family Health, School of Public Health, College of Medicine and Health Sciences, Wollo University, Dessie, Ethiopia; 2 Department of Epidemiology and Biostatistics, School of Public Health, College of Medicine and Health Sciences, Wollo University, Dessie, Ethiopia; FHI360, UNITED STATES

## Abstract

**Background:**

Closing the gap of unmet for family planning is crucial to eliminate new pediatric HIV infections likewise to improve maternal and child health among reproductive-age women living with HIV. However, studies conducted on unmet need for family planning among reproductive-age women living with HIV showed inconsistent and non-conclusive findings on the magnitude of the problem. Moreover, there was no meta-analysis conducted in this area. So this systematic review and meta-analysis were conducted to estimate the pooled prevalence unmet need for family planning among reproductive-age women living with HIV in Ethiopia.

**Methods:**

The Preferred Reporting Items for Systematic Reviews and Meta-Analyses (PRISMA) guideline was followed to review both published and unpublished studies in Ethiopia. All studies in PubMed, Cochrane Library, Hinari, Google Scholar, CINAHL, and Global Health databases were searched. Meta-analysis was performed using STATA 14 software. The heterogeneity and publication bias were assessed using the I^2^ statistics and Egger regression asymmetry test, respectively. Forest plots were used to present the pooled prevalence with a 95% confidence interval (CI).

**Results:**

This review included 7 studies, and 3333 study participants. The pooled prevalence of unmet need for family planning among reproductive-age women living with HIV in Ethiopia was 25.13% (95%CI: 19.97, 30.29). The pooled prevalence of unmet need for spacing and limiting was 13.91% (95%CI: 10.11, 17.72) and 9.11% (95%CI: 6.43, 11.78), respectively.

**Conclusions:**

One-fourths of reproductive-age women living with HIV had an unmet need for family planning. A variety of programmatic investments are needed to achieve more meaningful progress toward the reduction of unmet need for family planning among reproductive-age women living with HIV.

## Introduction

Women of reproductive age are disproportionately affected by the HIV/AIDS pandemic. In Sub-Saharan Africa (SSA), the region highly affected by HIV, women and girls continue to be the foremost affected and accounted for 59% of all new HIV infections in the region in 2019 [1]. In Ethiopia, women cover more than 60% and 55% of adults living and newly infected with HIV/AIDS, respectively [2]. Even though new HIV infections among children showed a dramatic decline (52%) from 2010 to 2019, still far to reach the 2020 targets set by The Joint United Nations Programme on HIV/AIDS (UNAIDS) and its partners [3, 4].

Family planning is one of the proven, cost-effective strategies for preventing vertical transmission of HIV. Studies have shown that even modest decreases in the number of pregnancies to HIV-positive women could prevent HIV-positive births at the same rates as the use of antiretroviral therapy (ART) for prevention of maternal to child transmission (PMTCT) [5–7]. In SSA, about 333,000 new infant infections could be averted annually, if all women in the region who did not wish to become pregnant could have access to contraceptive services [8, 9]. Providing universal access to contraception can also reduce maternal, infant, and child deaths by 40%, 10%, and 21%, respectively [10–12].

Despite this importance, about 270 million reproductive-age women (15–49 years) have an unmet need for contraception worldwide [13]. SSA has the highest prevalence of unmet need for contraception, where one in five women have an unmet need for spacing or limiting pregnancies [14]. An analysis of Demographic and Health Survey (DHS) data from 12 African countries other than Ethiopia showed that 9–23% of HIV-positive women had an unmet need for family planning [15].

There are different factors affecting unmet need family planning among reproductive age women living with HIV. These include; age [16–25], marital status [17, 20, 25], educational status [23], residence [24], monthly expenditure [18, 19], number of alive children [18, 24], number of desired children [24], intention to have children [24], history of contraceptive use [18, 23, 24, 26], HIV disclosure status [22, 24], duration on ART [22], knowledge on maternal to child transmission (MTCT) [16, 25], discussion on family planning with partner [16], and partner’s HIV status [21].

Reducing maternal death, ending HIV/AIDS epidemic, and ensuring universal access for family planning are key components of Sustainable Development Goals (SDGs), targets 3.1, 3.3, and 3.7 respectively [27]. Reducing the maternal mortality ratio (MMR) to 199 per 100,000 live births, HIV infection rate among infants less than 2%, and unmet need for family planning to 10% by 2020 are also some of the primary targets of the National Reproductive Health Strategy of Ethiopia [28]. Due to this, the family planning service has been given special attention by several governmental and non-governmental organizations.

Determining the prevalence of unmet need for family planning among reproductive-age women with HIV is important in designing effective interventions to reduce the problem. There is no nationally representative primary data source that provides an estimate of unmet need for family planning among HIV-positive women in Ethiopia. The available studies which assessed unmet need for family planning among reproductive-age women living with HIV in Ethiopia also revealed inconsistent and non-conclusive findings. Unmet need for family planning among women living with HIV in Ethiopia varied from 15.5% in Nekemet [16] to 35.3% in the Amhara region [25]. Therefore, this review aimed to estimate the pooled prevalence of unmet need for family planning among reproductive-age women living with HIV in Ethiopia.

## Methods

### Registration

This systematic review has been registered in the International Prospective Registry of Systematic Review(PROSPERO) with a specific registration number CRD42020155896.

### Reporting

Preferred Reporting Items for Systematic Review and Meta-Analysis (PRISMA) guideline was strictly followed in this review [29] (S1 Table).

### Search strategy

A systematic review and meta-analysis of published and unpublished studies were conducted to assess the pooled prevalence unmet need for family planning among reproductive-age women living with HIV in Ethiopia. Studies were searched through PubMed, Cochrane Library, Hinari, Google Scholar, CINAHL, and Global Health databases. Moreover, grey literatures were searched by tracing reference lists. The search was conducted from June 5–12, 2020. The search was made using the search term: "prevalence", "proportion", "magnitude", "incidence", "unmet need", "demand", "need", "family planning", "family planning utilization", "family planning use", "contraceptive use", "contraceptive utilization", "contraception", "factors", "determinants", "predictors", "factors associated", "associated factors", "risk factors", "women", "reproductive age women", "living with HIV/AIDS", "living with HIV", "HIV positive", "ART clinic, "ART care", "HIV/AIDS care", "Chronic HIV/AIDS care", "Ethiopia". All key terms were searched by a combination of Boolean operators “AND” or “OR” as appropriate and the search was done by two authors independently (BK and AA).

### Study selection and eligibility criteria

All available studies conducted from January 1, 2000, to June 1, 2020, which fulfilled the eligibility criteria were included in this review (Table 1).

**Table 1:** Eligibility criteria’s for studies include in meta-analysis of unmet need for family planning among reproductive-age women living with HIV in Ethiopia.

**Inclusion criteria**	Participants	Reproductive age women living with HIV
	Setting	Both community and institutional based studies
	Outcome	Unmet need for family planning
	Publication	Journal article, master thesis and dissertation
	Language	English
	Type of study	All observational study
**Exclusion criteria**	Studies conducted among male people.	
	Studies whichdid not report the outcome of interest.	

### Outcome measurements

#### Unmet need for family planning

The women were considered as having unmet need for family planning if they had unmet need for limiting and/or unmet need for spacing [30].

#### Unmet need for limiting

Sexually active woman who was not using any method of contraception, and did not want to have more children, and/or whose last pregnancy was unwanted and/or did not know whether to have children or not was taken as having unmet need for limiting.

#### Unmet need for spacing

Sexually active woman who was not using any method of contraception, and wanted to postpone their next birth for at least two years, and/or whose last pregnancy was mistimed and/or did not know when to have children was considered as having unmet need for spacing.

All studies which used the above definition to measure the prevalence of unmet need for family planning among reproductive-age women living with HIV were included in this review.

### Study selection, quality appraisal, and data extraction

Those articles searched from selected databases were exported to Endnote X8, and duplicate files were removed. The remaining articles and abstracts were independently screened by two groups (BA and MA) for inclusion in the full-text appraisal. The differences between reviewers were managed through discussion and disagreement was handled by the third party (MY). The quality of articles was assessed using the Joanna Briggs Institute (JBI) critical appraisal checklist [31]. Two reviewers independently assessed articles before inclusion for review.

Three authors (BK, YD, and MY) independently extracted all the necessary data using a using Microsoft excel 2010 sheet. The data extraction tool contains information on the author’s name, year of study, year of publication, study area, response rate, sample size, study quality score, and prevalence.

### Statistical methods and analysis

The meta-analysis was conducted using STATA 14 software. Forest plot was used to show the magnitude of unmet need for family planning among reproductive-age women living with HIV in Ethiopia. Due to the substantial presence of heterogeneity among studies, the random effect model of analysis was used. The pooled prevalence of unmet need for family planning was presented with 95% CI. The heterogeneity test of included studies was assessed by using the I^2^ statistics. It was declared using a p-value less than 0.05 for I^2^ statistics [32].

Subgroup analysis was also conducted by different study characteristics such as sub-region of Ethiopia (North or other), study year (during Millennium Development Goal period(MDG) period or Post MDG), study quality score (low or high score). The publication bias was assessed using the Egger regression asymmetry test [33, 34]. It was declared with a p-value of less than 0.05.

## Results

### Study selection

**Fig 1:**
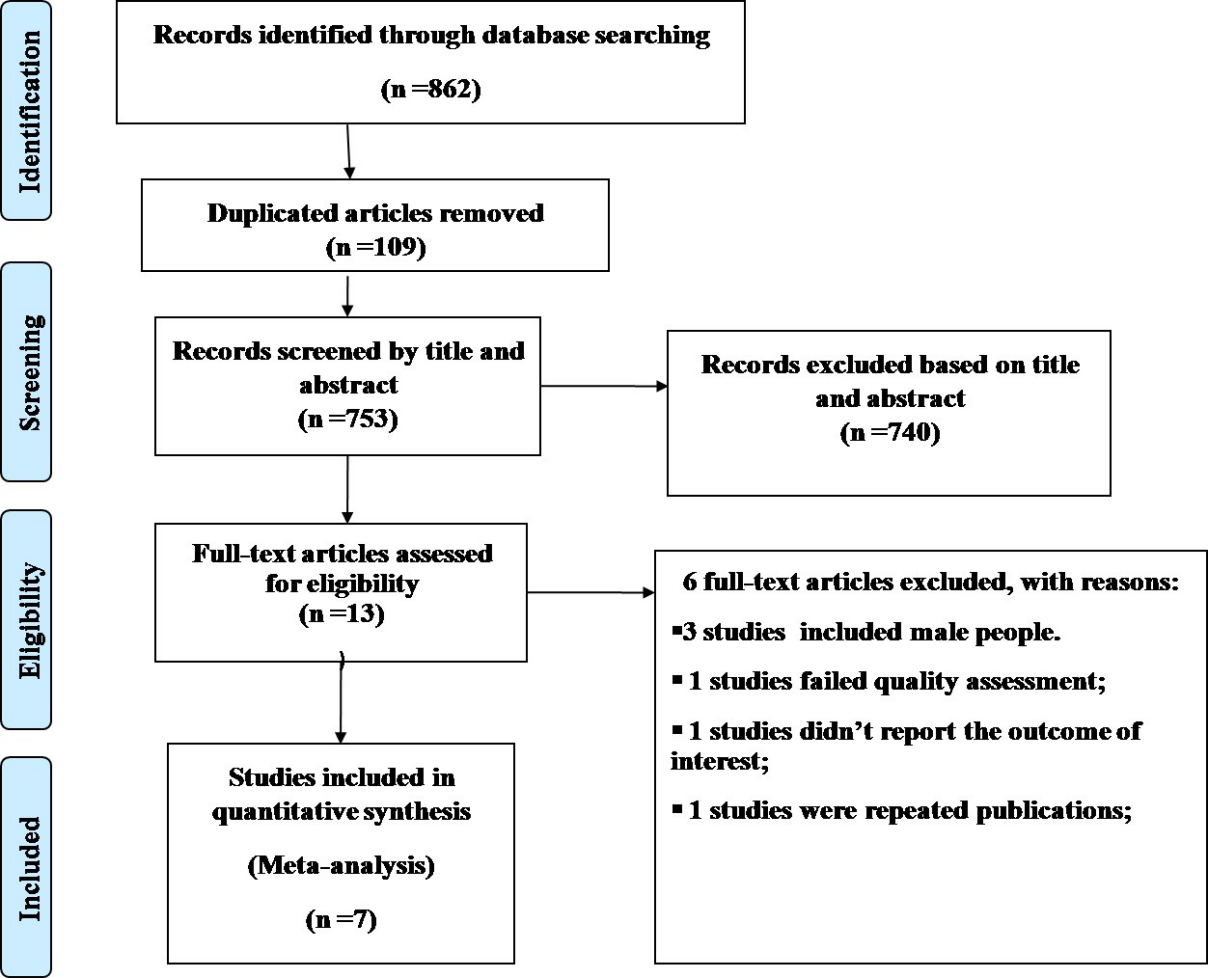
PRISMA flow diagram of the included studies for meta-analysis of unmet need for family planning among reproductive-age women living with HIV in Ethiopia.

This systematic review and meta-analysis included both published and unpublished studies conducted on unmet need for family planning among reproductive-age women living with HIV in Ethiopia. A total of 862 records were retrieved through electronic database searching. From these, 109 duplicated records were excluded, and the remaining 740 articles were excluded using their titles and abstracts. Thirteen full-text articles were assessed for eligibility. From these, 6 full-text articles were excluded for prior criteria [24, 35–39], and a total of 7 studies [16, 21–25, 40] were included in the review (Fig 1).

### Characteristics of included studies

Articles included in this review were both published and unpublished cross-sectional studies [16, 21–25, 40]. The sample size of studies ranged from a minimum of 334, a study conducted in Addis Ababa [21] to a maximum of 658, a study conducted in Hawassa [23]. A total of 3333 study participants were included in this review. The studies were conducted from 2013 to 2018 in different regions of the country. A total of four administrative regional states (Tigray, Amhara, Oromia and Southern Nations, Nationalities and Peoples’ Region) and one administrative city (Addis Ababa) were represented in this review (Table 2).

**Table 2:** Summary characteristics of studies included in the meta-analysis of the prevalence of unmet need among HIV positive women in Ethiopia.

Authors and Publication year	Study year	Study area	Study design	Sample size	Response rate	Prevalence (%)			Quality score
						unmet need for FP	Unmet need for Spacing	Unmet need for limiting	
Abeje and Motbaynor, 2016	2013	Amhara region	Institutional based cross-sectional	530	100	24.5	-	-	78%
Abubeker FA et al, 2019	2016	Addis Ababa	Institutional based cross-sectional	334	95.9	25.1	16.2	9.0	89%
Berhane K et al, 2018	2014	Tigray region	Institutional based cross-sectional	451	99.1	32.4	18.0	14.4	67%
Feyssa MD et al, 2015	2015	Hawassa City	Institutional based cross-sectional	658	99.6	19.1	13.2	5.9	89%
Feyissa and Melka, 2014	2014	Nekemte town	Institutional based cross-sectional	401	99.5	15.5	7.5	8.0	67%
Kassie MD et al, 2019	2018	Gondar city	Institutional based cross-sectional	441	100	24.5	15.4	9.1	78%
Zewdie Z et al, 2020	2018	Amhara region	Institutional based cross-sectional	518	100	35.3	-	-	78%

FP-Family Planning

### Prevalence of unmet need for family planning

**Fig 2:**
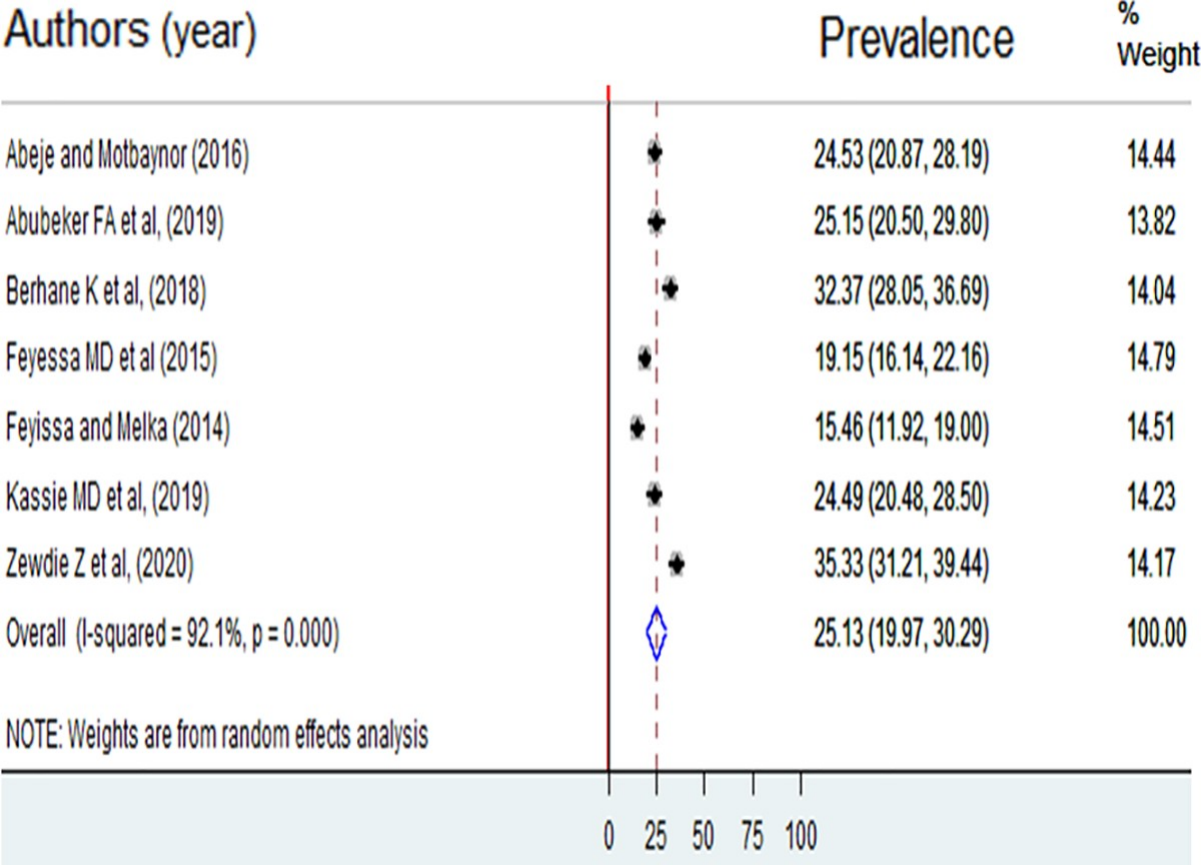
Prevalence of unmet need for family planning among reproductive-age women living with HIV in Ethiopia, 2013 to 2018.

The pooled prevalence of unmet need for family planning among reproductive-age women living with HIV in Ethiopia was 25.13% (95%CI: 19.97, 30.29). The highest prevalence of unmet need for family planning was reported from a study done in Amhara Region. The study showed that 35.3% of reproductive-age women living with HIV had an unmet need for family planning [25]. The lowest prevalence of unmet need for family planning was 15.5% among reproductive-age women living with HIV in Nekemte [16]. Substantial heterogeneity was found among included studies in the meta-analysis, I^2^ = 92.1%, and p < 0.001 (Fig 2). The funnel plot showed a symmetrical appearance. The Egger’s regression asymmetry test also showed non-significant publication bias, p-value = 0.35.

**Fig 3:**
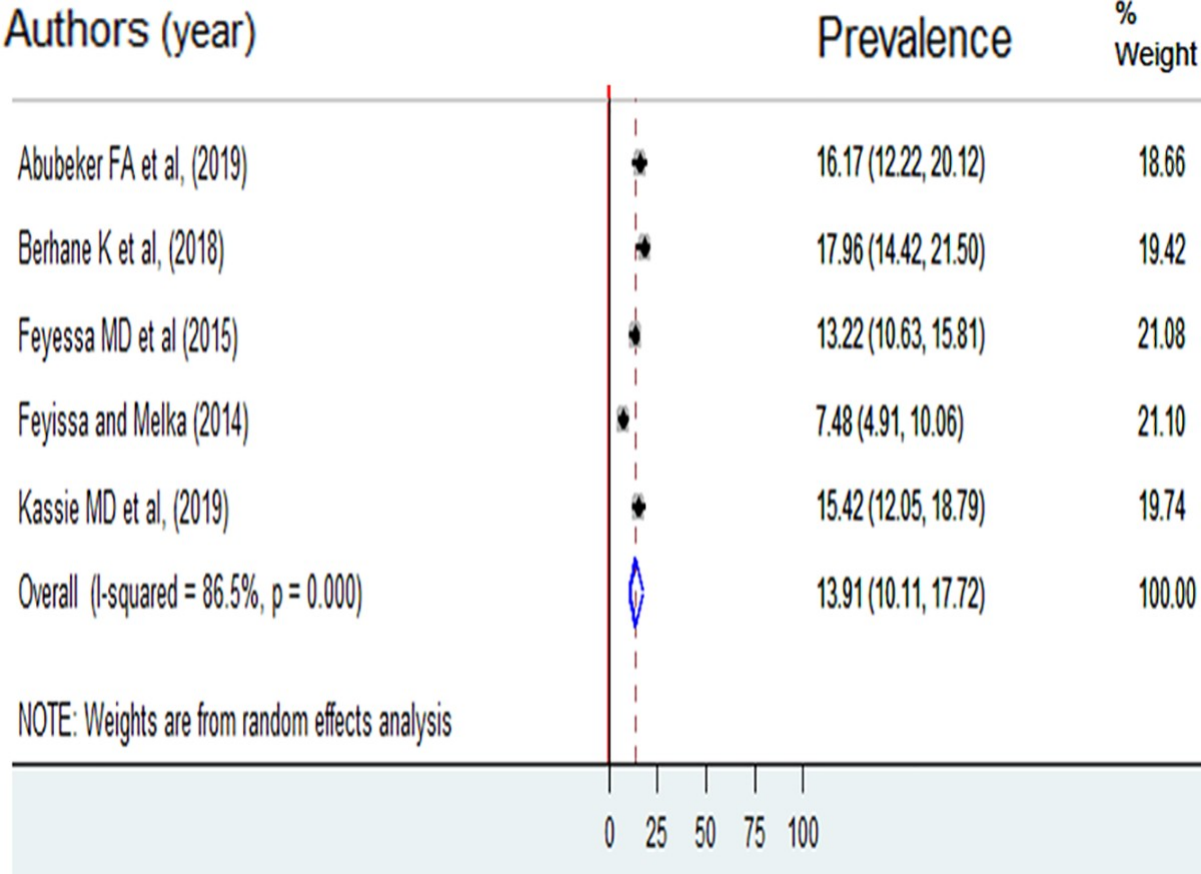
Prevalence of unmet need for spacing among reproductive-age women living with HIV in Ethiopia, 2013 to 2018.

**Fig 4:**
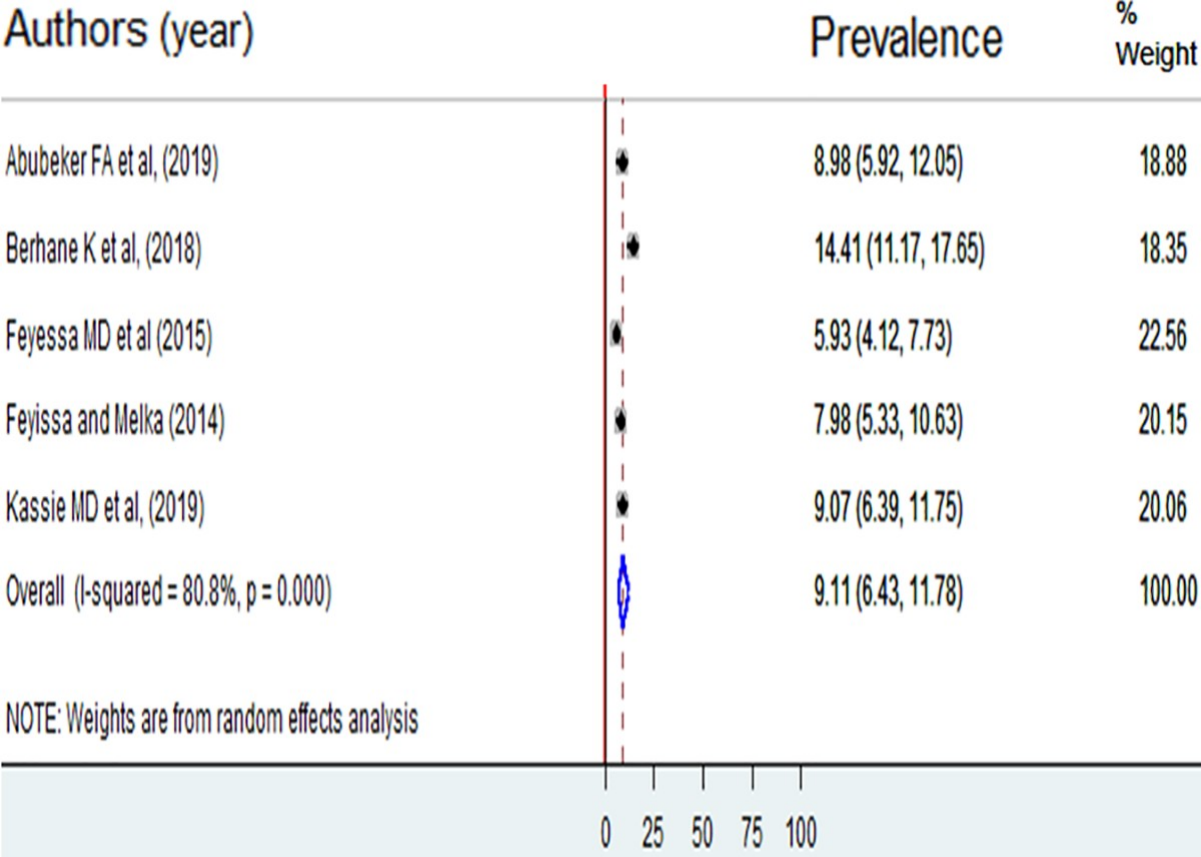
Prevalence of unmet need for limiting among reproductive-age women living with HIV in Ethiopia, 2013 to 2018.

Unmet need for spacing and limiting was reported by five of the included studies [16, 21–24]. The pooled prevalence of unmet need for spacing was 13.91% (95%CI: 10.11, 17.72) (Fig 3). The prevalence unmet need for limiting was 9.11% (95%CI: 6.43, 11.78) (Fig 4).

### Sub-group analysis

Sub-group analysis was conducted to deal with the source of heterogeneity. However, the heterogeneity still exists. Thus, the heterogeneity may be explained by other factors not included in this review. The prevalence of unmet need for family planning among studies done during and after 2015 was 25.96 (95% CI: 18.98, 32. 94). The prevalence of unmet need for family planning among studies done in the Northern part of Ethiopia was 29.12% (95%CI: 23.69, 34.56), which is higher than studies conducted in other parts of the country (Table 3).

**Table 3:** Subgroup analysis of the prevalence of unmet need for family planning among women living with HIV in Ethiopia, 2013–2018.

Sub-groups	Number of studies	Total sample	Prevalence (95% CI)	Heterogeneity	
				I^2^	p-value
By region					
North ^a^	4	1940	29.12 (23.69, 34.56)	86.3	< 0.001
Other ^b^	3	1393	19.70 (14.82, 24.58)	81.1	0.005
By quality score					
High	5	2481	25.65 (20.33, 30.97)	89.7	< 0.001
Low	2	852	23.87 (7.30, 40.44)	97.2	< 0.001
By study year					
Before 2015	3	1382	24.06 (14.66, 33.45)	94.5	<0.001
2015 and after	4	1951	25.96 (18.98, 32. 94)	92.3	< 0.001
Total	7	3333	25.13 (19.97, 30.29)	92.1	< 0.001

^a^ Tigray and Amhara

^b^ Addis Ababa, Oromia and Southern Nations, Nationalities and Peoples’ Region (SNNPR)

## Discussion

Unmet need for contraception is an important concept for designing family planning programs and has its own implications for maternal and child health, especially for reproductive-age women living with HIV. This systematic review and meta-analysis was conducted to estimate the prevalence of unmet need for family planning among reproductive-age women living with HIV in Ethiopia.

This study found a higher prevalence of unmet need for family planning among HIV-positive women in Ethiopia compared to other countries in Africa. The pooled prevalence of unmet need for family planning among reproductive-age women living with HIV in Ethiopia was 25.13% (95%CI: 19.97, 30.29). The DHS data analysis from 12 African countries also showed that nine countries had a level of unmet need for family planning lower than the finding of this study i.e 9.3% in Togo to 19.5% in Malawi, and other three countries Kenya (20.2%), Côte d’Ivoire (20.4%), and Togo (23.2%) had a similar level of unmet need for family planning with this review [15]. Most DHS included in the analysis were conducted before 2013, but all studies included in this review were conducted during and after 2013. It is also too far to achieve the United Nations Population Fund (UNFPA) consultation to end unmet need for family planning by 2030 [41]. Poor access and quality of family planning services disintegrated HIV/AIDS treatment and care services, absence of strong monitoring and follow-up system, and lack of networking and coordination might contribute to the high level of unmet need for family planning among reproductive-age women living with HIV in Ethiopia.

Studies conducted in the Northern part of the country had a higher level of unmet need for family planning (29.1%) than studies conducted in the other part of Ethiopia (Addis Ababa, Oromia, and SNNPR) (19.7%). The possible reason for this might be studies in the northern part of Ethiopia involved women from rural area higher than studies conducted in other parts of the country. Rural women have low knowledge and access for family planning services. Thus, women who reside in rural areas have a higher unmet need for family planning than women who reside in urban areas [42]. Furthermore, most studies in the other part of the country were conducted at hospitals. However, the majority of studies in the Northern part of the country were conducted at both hospitals and health centers. Hospitals had well equipped with human resources and materials, better health care delivery systems, and integrated health services than health centers.

Despite several efforts made by governmental and non-governmental organizations, the level of unmet need for family planning among HIV-positive women is still high. This review showed no reduction in the level of unmet need for family planning among HIV-positive women in Ethiopia in the years 2015 to 2018 (25.96%) [21, 23–25] compared with studies conducted before 2015 (24.06%) [16, 22, 40]. This calls for efforts to meet the need for family planning to achieve the SDG targets to end HIV/AIDS epidemics and to reduce maternal and child morbidity and mortality [27].

Family planning service is a promising strategy to reduce maternal and child morbidity and mortality by preventing high-risk and unwanted pregnancies. However, complications during pregnancy or childbirth are still one of the leading causes of death and disability among women of the reproductive age group in developing countries [43]. Therefore, emphasis should be given to reduce unmet need for family planning through the improvement of family planning access and choice, integration of family planning service with HIV treatment and care services, provision of HIV/AIDS patient-friendly health services.

This review has certain strengths and limitations. The PRISMA guideline was strictly followed in the systematic review and meta-analysis. Only four administrative regional states and one administrative city were included in the review. Thus, this may affect the representativeness of the review. Moreover, there were limited studies that presented factors associated with unmet need for family planning. The factors considered in these studies also vary across studies. For this reason, this review was unable to identify factors affecting the unmet need for family planning among reproductive-age women living with HIV.

## Conclusions

The prevalence of unmet need for family planning among reproductive-age women with HIV is high. One in four reproductive-age women living with HIV has an unmet need for family planning. The government and other concerned bodies should urgently stride to reduce unmet need for family planning through strengthening family planning programs and better integrate family planning services in HIV service delivery settings. Prong 2 of PMTCT (prevention of unintended pregnancy among HIV-positive women) must become more visible and given programmatic priority. It is also needed to transform services in the way that will help HIV-infected women and couples achieve their desired spacing, timing, and number of children. Moreover, further large-scale studies are also needed to investigate factors associated with unmet need for family planning among reproductive age living with HIV.

## Supporting information

S1 Table PRISMA-P 2009 checklist. (DOC)

S2 Table Quality assessment. (DOCX)

S1 File The search electronic strategy. (DOCX)

## Abbreviations

AIDS: Acquired Immune Deficiency Syndrome
ART: Antiretroviral Therapy
CI: Confidence Interval
DHS: Demographic and Health Survey
HIV: Human Immuno-deficiency Virus
JBI: Joanna Briggs Institute
PMTCT: Prevention of Maternal to Child Transmission
SDG: Sustainable Development Goal
UNAIDS: The Joint United Nations Programme on HIV and AIDS
UNFPA: United Nations Population Fund
