# Supplementary material for: Unmet need for family planning among reproductive-age women living with HIV in Ethiopia: A systematic review and meta-analysis
Source: PLoS One. 2021 Aug 2;16(8):e0255566. doi: 10.1371/journal.pone.0255566 (PMC8328287; doi:10.1371/journal.pone.0255566)
Supplement: S2 Table — (DOCX) [file pone.0255566.s002.docx]

**Table 3. Quality assessment for included studies**

|  | | **Articles** | | | | | | |
| --- | --- | --- | --- | --- | --- | --- | --- | --- |
|  | **Checklist** | Abeje G, et al, (2013) | Abubeker FA, et al, (2016) | Feyessa MD, et al, (2015) | Feyissa TR, et al, (2014) | Kassie MD, et al, (2018) | Zewdie Z, et al, (2018) | Berhane K , et al, (2014) |
| 1 | Was the sample frame appropriate to address the target population? |  |  |  |  |  |  |  |
|  | Yes | √ | √ | √ |  |  |  |  |
|  | No |  |  |  |  |  |  |  |
|  | Unclear |  |  |  | √ | √ | √ | √ |
|  | Not applicable |  |  |  |  |  |  |  |
| 2 | Were study participants sampled in an appropriate way? |  |  |  |  |  |  |  |
|  | Yes | √ |  |  |  | √ |  |  |
|  | No |  | √ |  | √ |  |  | √ |
|  | Unclear |  |  | √ |  |  | √ |  |
|  | Not applicable |  |  |  |  |  |  |  |
| 3 | Was the sample size adequate? |  |  |  |  |  |  |  |
|  | Yes |  | √ | √ | √ |  | √ | √ |
|  | No |  |  |  |  |  |  |  |
|  | Unclear | √ |  |  |  | √ |  |  |
|  | Not applicable |  |  |  |  |  |  |  |
| 4 | Were the study subjects and the setting described in detail? |  |  |  |  |  |  |  |
|  | Yes | √ | √ | √ | √ | √ | √ |  |
|  | No |  |  |  |  |  |  | √ |
|  | Unclear |  |  |  |  |  |  |  |
|  | Not applicable |  |  |  |  |  |  |  |
| 5 | Was the data analysis conducted with sufficient coverage of the identified sample? |  |  |  |  |  |  |  |
|  | Yes | √ | √ | √ | √ | √ | √ | √ |
|  | No |  |  |  |  |  |  |  |
|  | Unclear |  |  |  |  |  |  |  |
|  | Not applicable |  |  |  |  |  |  |  |
| 6 | Were valid methods used for the identification of the condition? |  |  |  |  |  |  |  |
|  | Yes | √ | √ | √ | √ | √ | √ | √ |
|  | No |  |  |  |  |  |  |  |
|  | Unclear |  |  |  |  |  |  |  |
|  | Not applicable |  |  |  |  |  |  |  |
| 7 | Was the condition measured in a standard, reliable way for all participants? |  |  |  |  |  |  |  |
|  | Yes | √ | √ | √ | √ | √ | √ | √ |
|  | No |  |  |  |  |  |  |  |
|  | Unclear |  |  |  |  |  |  |  |
|  | Not applicable |  |  |  |  |  |  |  |
| 8 | Was there appropriate statistical analysis? |  |  |  |  |  |  |  |
|  | Yes | √ | √ | √ |  | √ | √ | √ |
|  | No |  |  |  | √ |  |  |  |
|  | Unclear |  |  |  |  |  |  |  |
|  | Not applicable |  |  |  |  |  |  |  |
| 9 | Was the response rate adequate, and if not, was the low response rate managed appropriately? |  |  |  |  |  |  |  |
|  | Yes |  | √ | √ | √ | √ | √ | √ |
|  | No |  |  |  |  |  |  |  |
|  | Unclear | √ |  |  |  |  |  |  |
|  | Not applicable |  |  |  |  |  |  |  |
| IV. | **Result & Conclusion** |  |  |  |  |  |  |  |
|  | Result (sum of stars gained out of 9) | 7 | 8 | 8 | 6 | 7 | 7 | 6 |
|  | Conclusion of the quality of articles |  |  |  |  |  |  |  |
|  | Include | √ | √ | √ | √ | √ | √ | √ |
|  | Exclude |  |  |  |  |  |  |  |
