# Supplementary material for: Unmet need for family planning among reproductive-age women living with HIV in Ethiopia: A systematic review and meta-analysis
Source: PLoS One. 2021 Aug 2;16(8):e0255566. doi: 10.1371/journal.pone.0255566 (PMC8328287; doi:10.1371/journal.pone.0255566)
Supplement: S1 File — (DOCX) [file pone.0255566.s003.docx]

**S1 file. Searching strategy**

**PubMed**

((((((((((((((((((((((((((((Prevalence) OR (proportion)) OR (magnitude)) OR (incidence)) AND ("unmet need")) OR (demand)) OR (need)) AND ("family planning")) OR ("contraceptive use")) OR (contraception)) OR ("family planning utilization")) OR ("contraceptive utilization")) OR ("family planning use")) AND (factors)) OR (determinants)) OR (predictors)) OR ("associated factors")) OR ("factors associated")) OR ("risk factors")) AND (women)) OR ("reproductive age women")) AND ("living with HIV/AIDS")) OR ("living with HIV")) OR ("HIV positive")) OR ("ART clinic")) OR ("ART care")) OR ("HIV/AIDS care")) OR ("Chronic HIV/AIDS care")) AND (Ethiopia)

Filter applied: Specie-Humans, Language-English, Sex-Female, Age- (Adolescent: 13-18 years, Adult: 19-44 years, Middle Aged + Aged: 45+ years), and year- (January 1, 2000- June 1, 2020).

The search was made from 5-12 June, 2020.

**Global Health**

((((((((((((((((((((((((((((((((Prevalence) OR (proportion)) OR (magnitude)) OR (incidence)) AND ("unmet need")) OR (demand)) OR (need)) AND ("family planning")) OR ("contraceptive use")) OR (contraception)) OR ("family planning utilization")) OR ("contraceptive utilization")) OR ("family planning use")) AND (factors)) OR (determinants)) OR (predictors)) OR ("associated factors")) OR ("factors associated")) OR ("risk factors")) AND (women)) OR ("reproductive age women")) AND ("living with HIV/AIDS")) OR ("living with HIV")) OR ("HIV positive")) OR ("ART clinic")) OR ("ART care")) OR ("HIV/AIDS care")) OR ("Chronic HIV/AIDS care")) AND (Ethiopia)) AND yr:[2000 to 2020]

Refined by: Language- English; Geographical location-Ethiopia; Topic; Family planning and women

**Cochrane Library**

((((((((((((((((((((((((((((Prevalence) OR (proportion)) OR (magnitude)) OR (incidence)) AND ("unmet need")) OR (demand)) OR (need)) AND ("family planning")) OR ("contraceptive use")) OR (contraception)) OR ("family planning utilization")) OR ("contraceptive utilization")) OR ("family planning use")) AND (factors)) OR (determinants)) OR (predictors)) OR ("associated factors")) OR ("factors associated")) OR ("risk factors")) AND (women)) OR ("reproductive age women")) AND ("living with HIV/AIDS")) OR ("living with HIV")) OR ("HIV positive")) OR ("ART clinic")) OR ("ART care")) OR ("HIV/AIDS care")) OR ("Chronic HIV/AIDS care")) AND (Ethiopia)

**CINAHL**

(((((((((((((((((((((((((((((((Prevalence) OR (proportion)) OR (magnitude)) OR (incidence)) AND ("unmet need")) OR (demand)) OR (need)) AND ("family planning")) OR ("contraceptive use")) OR (contraception)) OR ("family planning utilization")) OR ("contraceptive utilization")) OR ("family planning use")) AND (factors)) OR (determinants)) OR (predictors)) OR ("associated factors")) OR ("factors associated")) OR ("risk factors")) AND (women)) OR ("reproductive age women")) AND ("living with HIV/AIDS")) OR ("living with HIV")) OR ("HIV positive")) OR ("ART clinic")) OR ("ART care")) OR ("HIV/AIDS care")) OR ("Chronic HIV/AIDS care")) AND (Ethiopia)

Refined by: Year- January, 2000- June 2020; Age- (13-18 years, 19-44 years, 45-64 years); Journal subset- (Public health, Health promotion/education)

**Hinari**

((Prevalence) OR (proportion) OR (magnitude)) AND (("unmet need") OR (need) OR (demand)) AND (("family planning") OR ("contraceptive use") OR ("contraceptive utilization") OR ("family planning utilization")) AND ((factors) OR (predictors)) AND ((women) OR ("reproductive age women")) AND (("living with HIV") OR ("HIV positive") OR ("ART clinic")) AND (Ethiopia)

Refined by: Content type- Journal article and article; Date of publication- January 1, 2000- June 1, 2020; Discipline- Medicine and public health; Subject term- female; and Language-English

**Google Scholar**

In Google scholar database the following key terms were searched by a combination of Boolean operators “AND” or “OR” as appropriate.

"prevalence", "proportion", "magnitude", "incidence", "unmet need", "demand", "need", "family planning", "family planning utilization", "family planning use", "contraceptive use", "contraceptive utilization", "contraception", "factors", "determinants", "predictors", "factors associated", "associated factors", "risk factors", "women", "reproductive age women", "living with HIV/AIDS", "living with HIV", "HIV positive", "ART clinic, "ART care", "HIV/AIDS care", "Chronic HIV/AIDS care", "Ethiopia".
